# Warm and Hot Water as a Surgical Dressing

**Published:** 1879-04

**Authors:** Frank H. Hamilton

**Affiliations:** Wards at Bellevue Hospital


*ART. II.—Warm and Hot Water as a Surgical Dressing.* Resumé of cases treated in Dr. FRANK H. HAMILTON'S Wards at Bellevue Hospital, November and December, 1878; with supplementary remarks.

The object of this paper is to show, in some degree, the range of usefulness of warm and hot water in the treatment of surgical accidents; and although a service of two months, even in a large hospital, is too short to demonstrate its value in many suitable cases, yet it will be found long enough to teach a good many useful lessons.

CASE 1.—*Lacerated Wound of Arm.* Victor Cavart, æt. 19, November 12, 1878, his arm was caught in the cog-wheels of some machinery, and being admitted to ward 11 on the same day, there was found to be a badly lacerated wound of half the length of the forearm. Hot water fomentations were at once applied, as there was no bleeding. On the 14th the bath was substituted. 19th, fomentations resumed. 25th, water dressings were discontinued, (14th day.) Dec. 7th, left the hospital, but returned occasionally to have it dressed. Dec. 28th, (less than seven weeks,) cicatrization complete.

In this case, although there was extensive contusion with the laceration, very little inflammation, and scarcely any destruction of tissue by gangrene ensued.

CASE 2.—*Traumatic Erysipelas.* Michael C. Broderick, æt. 52, driver, fell from his truck Nov. 25th, 1378, inflicting a severe lacerated and contused wound upon his forehead. The wound extended to the bone. He was admitted to the hospital on the same day, but not until some physician had very improperly sewed up the wound. Although the stitches were removed within twelve hours after they had been inserted, the neighborhood of the wound and the side of the face had already become red and swollen. Pus soon formed beneath the skin, and on the 9th day an erysipelatous inflammation extended over a considerable portion of the side of the temple and face.

At this time, on the 9th day, my attention was called to the patient, and I ordered water fomentations; temperature of about

100° Fahrenheit. Nine days later the erysipelas had disappeared and the warm water was discontinued, no other treatment, either local or constitutional having been employed. I may add that I have not for several years (probably fifteen years) adopted any other treatment in traumatic erysipelas, and no other *local* treatment in any other form of erysipelas, either in private or hospital practice; that in no instance has the erysipelas extended, except very slightly, while the water was continued, and all have recovered; and, so far as I can judge, as rapidly as they could have recovered under any other plan of treatment.

Case 3.—*Autopsy Wound. Septicæmia.* James O'Brian, æt. 40, employed in the Dead-House, while sewing up a body upon whom an autopsy had just been made, pricked the middle finger of his left hand just above the last phalangeal articulation. This occurred on the 6th of November, 1878. Within twenty-four hours the whole hand became swollen, red, hot and throbbing, the pain extending up to the axilla and breast. Forty-eight hours after the accident he consulted Dr. McC., who ordered a dose of castor oil and a flax-seed poultice. The swelling continued to increase.

O'Brian was admitted to the hospital on the fourth day after the injury was received, with his hand and arm much swollen to near the elbow, and with considerable febrile disturbance. He was much depressed from apprehension. On admission he was assigned to ward No. 1, by mistake, where he remained four hours, when the error was corrected and he was transferred to ward 30. Within an hour hot water fomentations were applied to hand and forearm, including the elbow, by my chief of staff, Dr. Hochheimer, who ordered also a dose of castor oil. Within an hour he declared himself relieved of the pain; and on the 16th of November, sixteen days after admission, the swelling of the hand and forearm had entirely disappeared and the water dressing was discontinued. He was discharged cured on the 20th of November. He has since informed me that the flax-seed poultice gave him no relief, but that the warm water fomentations brought relief almost at once.

Case 4.—*Chronic Ulcers of Leg. Granulations freshened by*

*hot water; subsequent successful Grafting.* T. F., æt. 24, admitted Dec. 12, 1878, with an extensive ulcer of the leg, which had existed three years. The ulcer was indolent, and was discharging a thin sanies. The loss of skin was so extensive that it was decided at once that by grafting alone could we succeed in covering the whole surface with new skin; but preparatory to the grafting it was necessary to render the granulations healthy. Accordingly, the patient having been put in bed, hot water fomentations were applied, and on the fifth day the granulations were red, firm and in all respects healthy. We at once proceeded to place upon the surface quite a number of small grafts, nearly all of which became adherent, and two weeks later the ulcer was closed.

This patient had come to me from Illinois, representing that he had never been able to make the sore heal since the skin was first destroyed by a severe injury. The result illustrates, therefore, both the value of hot water in revivifying indolent granulations, and also the value of skin grafting in this class of intractable cases.

I regret to be obliged to add that about a month later, while the patient was sitting up, the new skin again ulcerated in several points, and the hot water treatment had to be resumed.

Case 5.—*Indolent Ulcer of Foot; granulations encouraged by hot water, followed by successful Grafting.* Pat. Bannon, æt. 32, suffered a laceration, followed by sloughing of the integument over a considerable extent of one foot. The accident happened in June, 1878, and about six months later the wound had not healed. November 25th, 1878, he was ordered to bed and hot water fomentations applied. Five days later the granulations looking fresh and red, the ulcer was grafted at several points. On the 5th of December, many of the grafts had taken, and on the 1st of January, 1879, the cicatrization was complete for the first time since the accident occurred. Five weeks from the day on which the hot water applications were commenced. It must not be understood that the hot water was continued after the grafts were applied. I think the dressings after this were adhesive strips and Balsam of Peru. My only purpose in reporting this case, therefore, as in the preceding case, is to show how speedily the granulations were brought into a condition to bear the grafting. Grafts never do well on unhealthy ulcers.

Case 6.—*Compound Fracture of Tibia and Fibula. Gangrene and Erysipelas arrested by hot water. Spontaneous separation and recovery.* Robert Edgerton, æt. 41, sustained a compound fracture of both bones of the left leg above the ankle, on Dec. 13th, 1878. He was taken to Roosevelt Hospital, and the limb was dressed according to Listers method. The patient refusing to remain, was sent home, and on the 16th, he was sent to Bellevue Hospital, ward 11; when admitted his pulse was 134, and his temperature 103. The whole limb below the point of fracture was in a state of gangrene, and an erysipelatous blush extended far up the limb. His condition did not warrant a reasonable hope that amputation would save his life; and in case an amputation were made, it could not be made with any hope of success at a point lower than the knee-joint.

The limb was immediately enveloped in hot water fomentations at a temperature of about 105° or 110° F.

December 20th, the erysipelatous blush had receded and a line of demarcation had formed in the soft parts near the seat of fracture.

December 23d, with forceps and scissors I removed the dead limb at the seat of fracture, or a little below, without the loss of a drop of blood. Hot water continued.

January 1st, 1879. Hot water discontinued, wound granulating well. Patient in good condition. Substituted vasaline dressings for the hot water.

Case 7.—*Trephining Tibia. Treatment of the Wound with Warm Water.* J. T., æt. 22; admitted to Ward 5, Nov. 1878, suffering from ostitis (*osteo-myelitis*) of the lower end of the tibia; bone expanded and tender; suffering from severe and almost constant pain. In order to relieve the inflamed and imprisoned tissues, but not with the expectation of finding pus, I trephined the tibia before the class of medical students.

Warm water fomentations—temperature of about 95° or 100° F.,—were at once applied, and continued uninterruptedly until the wound had filled with granulations and cicatrization was nearly complete. From the date of the operation the pain ceased, and the cicatrization was effected in about two or three weeks.

CASE 8.—*Amputation of first Metacarpo-phalangeal Bone.—Neuritis.* Margaret Mahar, æt. 35, had a felon on right ring finger when 10 ten years old, which was opened, and left some contraction of the finger.

When 25 years old she had a felon on her right thumb, resulting in the loss of the last phalanx.

In 1877, a felon occurred on the right index finger; an incision made for its relief resulted in a tedious ulceration, and finally a cicatrization with contraction of the finger at a right angle.

June, 1878, she was admitted to Bellevue Hospital with a broken rib; and on August 10th (service of Dr. Mott) to relieve the deformity, the index finger was amputated through the middle of the first phalanx, taking care to remove all of the old cicatrix, which was very sensitive.

The sensitiveness remained after the healing of the stump; and, indeed, it was rather increased. Sept. 4th (service of Dr. Mason), the finger was disarticulated at the metacarpo-phalangeal articulation. In this operation, and during the subsequent treatment of the wound, Lister's method was employed; but, as the wound healed, the pain and sensitiveness increased.

Dec. 12th, the pain in the stump not having abated, and the patient desiring that another attempt should be made to give her relief—and she then being in my service—Dr. Hockheimer, my chief-of-staff, made at my request another amputation, removing the metacarpal bone at its articulation with the trapezium. All of the cicatricial tissue was carefully removed.

The wound was not closed by sutures, as in both of the preceding operations, nor were any ligatures required; but it was at once dressed with warm water fomentations, and these were continued until the cicatrization was nearly completed. The edges of the wound became infolded considerably during the progress of the cicatrization, and this suggested to some of those who saw it at this stage, that it would be necessary to dissect up the infolded skin and unite their edges by sutures. I did not predict that this would become necessary, and the result proved that it was not. The inverted skin gradually unfolded itself, and the wound healed without any crease or depression at the point of union.

I would have been glad to be able to say, both for the good of the poor woman and for the reputation of warm water, that the nervous sensibility and pain did not return, but they did return, yet somewhat tardily. I cannot say positively whether, in this respect, she is better or worse than she was before the last operation, but probably about the same. The fact is, I fear, that she has become affected with a chronic neuritis, for which, in my experience, neither re-amputation or resection of nerves has ever yet brought permanent relief. A cure may sometimes be effected by other means, but not by these.

Case 9.—*Acute Rheumatic Arthritis.—Relative Effects of Cold and of Hot Watar, in the same case.* Mary Welch, æt. 42, admitted to Ward 12, Sept. 4th, 1878, Dr. E. Mason's service. About the 21st of August preceding she had been seized at night with a severe pain in the right wrist, followed by pain, heat and redness, accompanied with chills, fever and sweat. Two weeks after admission to the Hospital the inflammation left her wrist, and a week later it attacked the left knee, which became swollen and very painful; the leg becoming gradually contracted—flexed—upon the thigh. Oct. 2d, extension was applied with 4½ pounds, and on the 5th the weight was increased to 9 pounds., and pads placed under the knee. Oct. 6th, limb laid on a double inclined plane, and extension continued. Oct. 9th, severe abdominal pains; apparatus, etc., removed and limb supported by pillows; after which the pain soon disappeared. Oct. 11th, extension again applied with 4½ pounds; limb supported by pillows.

Oct 19, *ice bags* applied to knee; extension continued. The ice was continued three days, and then Tinct. Iodine was applied. Nov. 7th, ice bag applied again, and continued to Nov. 16th (nine days), and the extension to the 15th (more than one month).

I had been on duty since the first of Nov., and as the limb was now straight, and the application of the ice bags was agreeable to the patient, I had not interfered with the treatment; but on this day I made a careful examination, in order to determine whether the patient might not leave the bed. I found the knee straight, and without swelling, but almost completely anchylosed; while the slightest attempt to move the joint caused most exquisite pain. Directing my attention to the ankle, I observed that the long continued extension had brought the foot down, with the toes pointing to the foot of the bed; and that in this position it was, also, almost completely anchylosed, and the slightest attempt to elevate the foot caused exquisite pain through the ankle and tarsus.

I will now say, unhesitatingly, that the ice-bags and the extension were responsible for the anchylosis both at the knee and ankle, and also for the exquisite sensibility of these parts when the limb was moved. These means had subdued the swelling of the knee, and straightened the leg upon the thigh; but they had, at the same time, caused the anchylosis and the morbid sensibility of the knee, and had distorted and anchylosed the foot and rendered it, also, equally sensitive to motion; although at the foot, so far as we know, no disease had previously existed. The ice-bags had also, I believe, given rise to a similar morbid sensibility, which extended over the integument of the whole limb below the knee. This is no new experience to me. I have seen this morbid sensibility induced by the long continued application of ice-bags before, and especially when they have been applied in persons of a feeble constitution, and nervous temperament. Others have observed the same. Essentially this opinion is expressed by Sanson, when he says: "It is well known that cold applications may cease to be useful, and may even become hurtful, by rendering the flesh oedematous, and pale, and causing it to become irritable when suppuration is established in the wounds." Cloquett remarked to M. Amussat (who is my authority for the above statement), that "he had observed the phenomena noticed by Sanson, in debilitated subjects, when cold has been used perseveringly." I will add, also, the testimony of a gentleman who 25 or 30 years ago was the leading Surgeon of Northern New York, and whose reputation as a man of large experience and careful observation was known throughout the State—Dr. Amasa Trowbridge, of Watertown, and Prof. of Surgery in the Willoughby Medical College, Ohio. In a letter to me he took occasion to speak of what he termed the injudicious practice of some Surgeons in using cold water too freely in the case of sprains, which had often, in his experience, induced a permanent stiffness of the joints, and rendered them sensitive and painful.

But it will be unnecessary to sustain my statement as to the cause of the anchylosis and sensitiveness in this case, by referring to the opinions of others, inasmuch as the result of the change which was made on the 16th will probably supply all the testimony which is needed.

On the morning of this day, the 16th, the ice-bags were removed from the knee and foot (to which latter they had been applied for only a short time), and at night the hot water fomentations were substituted. These were found to be more comforting to the patient than the ice-bags; from day to day the sensibility of the skin and of the joints steadily and rapidly diminished, while there was no return of swelling; the anchylosis yielded in the same degree, and on the 5th of December, less than three weeks from the date of the application of the hot water, she was walking on crutches, and her joints were quite flexible. Of course this was not accomplished without giving daily passive motion to the joints, and very gentle massage was also employed: but neither of these were possible, as we demonstrated by trying, until the hot water had been applied. Before this the patient would not bear the touch of a finger upon the skin, or the feeblest possible motion of the joints without crying out with pain.

I have dwelt thus long upon the history of this case, because its lessons seem to be very instructive, and may prove useful in their application to many other analagous cases.

*Brief Synopsis of the Principal Methods of Using Warm and Hot Water in Surgical Diseases and Accidents.* We usually speak of water as being "cold" when the temperature is below 68° F.; as "tepid" when the temperature is between 77 and 86° F.; as "warm" when it is between 90° and 100° F.; and as "hot" when it is above 100° F.; but, of course, the application of these terms is somewhat indefinite, since what may be warm to one may seem hot or tepid to another. It will be understood, however, distinctly that in speaking of warm water I do not mean tepid.

Water in the form of a continuous *bath* is in general more efficient than when used in the form of fomentations; but its application is necessarily, in most cases, limited to the hand, forearm, foot and leg. When employed in the case of lacerated and contused wounds it ought not to be continued much, if at all, beyond the period of acute inflammation—perhaps we may say from seven to ten days; and even during this period, if the patient is incommoded by the bath, the limb may occasionally be removed and treated by fomentation. If continued too long in the bath the limb is apt to become oedematous. No limb ought to be put in a warm or hot bath until all bleeding has ceased, and a delay of twelve or even twenty-four hours does not abate its good results. In these cases no sutures, straps or bandages are useful, but on the contrary, they are generally injurious.

Mr. John Rynders, of 304 Fourth Avenue, New York, has made under my instructions some zinc arm and foot baths for this purpose; but in almost any private house very excellent substitutes may be found.

For the purpose of *fomentations* we may use one or two thicknesses of sheet lint saturated with the warm or hot water, and applied closely to the entire circumference of the limb, the whole being wrapped in oiled silk cloth. To be most effective the sheet lint must be made to touch the surface of the skin, and open wounds, everywhere, and it must extend some distance beyond the parts injured; the oiled silk must be opened as often as every half hour, and the water again applied so as to keep up the elevated temperature; but the dressings need not be renewed oftener than once or twice daily, since the constant re-application will drain off the discharges. Of course this plan renders necessary some arrangement for drainage—and there are many cases in which the water can not be used thus freely, because the drainage cannot be made so complete. We thus lose some of the advantages, but the method will not be without its good results.

Cotton batting may be used as a substitute for sheet lint, especially that which is deprived of its oil, and is known now as the "absorbent cotton."

In the ear it is used as a bath, by pouring it into the ear; in the eye as a bath, effected by means of an eye cup, or by fomentations; in the nose by insufflation; in eczema and herpes, as a bath or fomentation; in the vagina as a douche, and as a means of arresting hæmorrhage, by the sponge.

*Summary of the Surgical Diseases and Accidents to which Warm and Hot Water may be Advantageously applied.* I shall enumerate first only those in which my own experience has demonstrated their usefulness.

1. Contusions, sprains, acute and subacute synovitis, subacute rheumatic arthritis, anchylosis of joints due to passive contraction of ligaments, chilblains. (Hot water.)

My experience in the use of hot water as a remedy in chilblains is limited to one trial in my own person. I have frequently suffered from chilblains in my toes during the colder seasons of the year, and I have always been able to obtain relief by immersing the foot in cold water for a few minutes, but during the present unusually severe winter one of my toes having become swollen and painful I determined to try hot water. During the two preceding nights I had been very much disturbed by the constant burning pain. In the morning I put my foot into a basin of water at a temperature of about 100° F. At first and for about two minutes this caused severe, sharp pains, like the pricking of needles, when the pains suddenly subsided; but at every attempt to elevate the temperature the pricking pains would return, and then again subside. I repeated this several times, and always with the same result. When I removed my foot it was quite easy, and on the following day the swelling had also disappeared, and neither the pain nor swelling have returned. The pricking sensation I ascribe to the returning circulation, by which the congestion or stasis of the blood in the part is overcome and the cure is effected. This happens, also, when cold water is temporarily applied, but only by the reaction which ensues. Hot water effects directly and instantly, what cold water effects indirectly and more slowly. Whether for this reason hot water is any more efficient or more permanently effectual than cold water, I am unable now to say.

2. *Lacerated and Contused Wounds, Punctured, Poisoned and, Gunshot.* (Warm or hot.) It is not generally prudent to employ water in these cases, until the bleeding has ceased; and especially may it be necessary to delay the use of the bath under these circumstances for a few hours.

3. *Traumatic Gangrene.* (Hot water.) While the curative effects of warm or hot water have been in many other cases surprising, it is in this class of cases that the effects of hot water have been to me wonderful. Many of these cases, in which limbs and lives have been saved when no other surgical expedient seemed to offer any chance, have from time to time been published by me in the *Medical Journals.*

4. *Erysipelas, especially Traumatic.* (Warm or hot).

5. *Indolent, Inflamed and Stoughing Ulcers.* (Warm or hot).

6. *Otitis Media.* I have seen my friend, Dr. St. John Roosa, oculist and aurist of this city, in a case of acute Otitis Media, attended with acute pain, pour into the ear with a teaspoon a few drops of water as hot as it could be borne, and after three ar four repetitions, within half an hour, the pain was relieved and the inflammation speedily abated. He informed me that this is his first resort in all of these cases.

7. *Parenchymatous Hæmorrhage, from the small vessels after amputation, and in all wounds.* (Very hot water). For a long time I have observed, and others have observed before me, that water, at almost a boiling temperature, applied by the aid of a sponge, held in a pair of forceps, will arrest very speedily a troublesome parenchymatous hæmorrhage. Cold water or ice may do the same, but the cold acts in a great measure by forming clots in the vessels, and when reaction takes place the bleeding may return; but hot water shuts up the vessels and is less liable to be followed by secondary hæmorrhage, and is never likely to prevent union by first intention. The spray of carbolic acid does the same, and this is one reason why it is serviceable; but in this respect it is no better than hot water.

8. *Eczema and Chronic Herpes exedens, especially in the Chronic Eczema of the Legs, of the Verge of the Anus and Vulva.* (Hot water). Dr. Lucius O. Bulkley, dermatologist of this city, and others, have publicly affirmed their confidence in its value in these and other similar cases.

In addition, I refer to the experience of Dr. A. H. Goelet, of this city, as to the value of hot water fomentations, with bandages, in the treatment of Phlegmasia Dolens, in one of the late numbers of the *New York Hospital Gazette.*

There is some recent testimony as to the value of warm water in traumatic tetanus, also; although in two cases in which I have tried it, it has failed. Upon this point, and as to its applicability to the gangrena senilis, and allied affections. my experience has not yet been sufficient to warrant the expression of an opinion.

Hot water injections have also, in my experience, and in the experience of many others proved exceedingly useful in various vaginal and uterine affections. Dr. Pitcher, of Detroit, and Dr. Emmett, of this city, were the first among the Surgeons of the present day to call attention to this fact; and most gynaecologists in this country are now using the injections satisfactorily.

Dr. C. Agnew, the well known opthalmologist of this city, uses hot water in certain corneal irritations and chronic granulations.

Dr. Leonard, of Detroit, uses hot water with salt, in acute nasal catarrh, by the douche.

*Accidents which, in my experience, Warm and Hot Water will Prevent.* They prevent traumatic erysipelas, septicaemia and gangrene; at least I may say, that not in one of hundreds of cases of traumatic injuries treated under my observation, have either of these accidents or sequelæ occurred, when warm or hot water have been employed from the first.

*Finally.* There is not much in all this which is new. Hippocrates recommended hot water in "fractures of the bones. especially of those which have been exposed," (compound); in "wounds of the head and in mortifications ; in herpes exedens of the anus, of the privy parts, the womb, the bladder." * * * Speaking of dislocations of the ankle he says: "At each time that the bandages are taken off, much hot water is to be used, for in all injuries at joints the affusion of hot water in large quantities is to be had recourse to." Hot water, he says, is anodyne. It soothes rigors, spasms and tetanus. It is useful "in pains, suppurations, frequent tears, and deep ulcers of the eyes."

Celsus, Galen and Rhazes of the older writers, repeated very much the same sentiments. In 1732, Lamorier called attention to the use of warm water. Guyot. Louis, Paré and Guthrie have added their testimony.

In Germany the value of warm water has been extolled by Max Schede and by Billroth.

In this country, and within a few years, warm or hot water have been used satisfactorily in one way or another (I refer only to those whose opinions have been publicly recorded), by Moses Gunn, of Chicago, E. M. Moore, of Rochester, by my late pupils Fred. E. Hyde, of New York (see this journal Dec. and Jan., 1875–6), and A. H. Goelet, also of this city, by C. Henri Leonard of Detroit, Mich., the late Paul F. Eve, of Nashville, Tenn., by Emmett, Agnew, Piffard, Roosa and Bulkley, of this city.

Of the verbal testimony of Surgeons brought to me from numerous sources, I do not feel at liberty to make a public statement.

P. S (March 20, 1879).—In case 6 I made a resection of the protruding bone in February, a sharp erysipelas followed, occasioned probably by the Esmark bandage; the erysipelas commencing six or eight inches above the end of the stump, where the pressure of the bandage was greatest. This is the first case in which I have known erysipelas to commence while warm water was being used; but under the continued application of the water it soon disappeared, and is now healing rapidly.

